# Twitter: A Novel Tool for Studying the Health and Social Needs of Transgender Communities

**DOI:** 10.2196/mental.4113

**Published:** 2015-05-07

**Authors:** Evan A Krueger, Sean D Young

**Affiliations:** ^1^ Department of Community Health Sciences University of California, Los Angeles Los Angeles, CA United States; ^2^ UC Institute for Prediction Technology Department of Family Medicine University of California, Los Angeles Los Angeles, CA United States

**Keywords:** Twitter, social media, transgender, health

## Abstract

**Background:**

Limited research has examined the health and social needs of transgender and gender nonconforming populations. Due to high levels of stigma, transgender individuals may avoid disclosing their identities to researchers, hindering this type of work. Further, researchers have traditionally relied on clinic-based sampling methods, which may mask the true heterogeneity of transgender and gender nonconforming communities. Online social networking websites present a novel platform for studying this diverse, difficult-to-reach population.

**Objective:**

The objective of this study was to attempt to examine the perceived health and social needs of transgender and gender nonconforming communities by examining messages posted to the popular microblogging platform, Twitter.

**Methods:**

Tweets were collected from 13 transgender-related hashtags on July 11, 2014. They were read and coded according to general themes addressed, and a content analysis was performed. Qualitative and descriptive statistics are presented.

**Results:**

There were 1135 tweets that were collected in total. Both “positive” and “negative” events were discussed, in both personal and social contexts. Violence, discrimination, suicide, and sexual risk behavior were discussed. There were 34.36% (390/1135) of tweets that addressed transgender-relevant current events, and 60.79% (690/1135) provided a link to a relevant news article or resource.

**Conclusions:**

This study found that transgender individuals and allies use Twitter to discuss health and social needs relevant to the population. Real-time social media sites like Twitter can be used to study issues relevant to transgender communities.

## Introduction

### Difficulties in Studying Transgender Communities

Limited research has been conducted that aims to better understand the shared experiences of transgender and gender nonconforming individuals. Despite an increasing interest in transgender health issues, researchers and practitioners have struggled to conduct studies that are representative of this diverse and heterogeneous population. Transgender individuals may not be identifiable, and may take action to remain hidden in public spaces [1], impeding study recruitment through traditional means. Much research to date has relied on clinic-based sampling methods. This approach may oversample transsexual individuals, who commonly seek medical intervention [2]. Doing so may bias findings about the most salient health needs of transgender communities as medical in nature.

Despite the diversity inherent to the transgender population, individuals often share common experiences of violence, harassment, and discrimination [3-5]. Transgender youths are more likely to drop out of school, report difficulty finding employment, and report engagement in sex work, compared to the general population [6-8]. Such experiences lead to elevated rates of mental and psychological distress [9,10], as well as decreased rates of health care utilization [11], likely due to perceived stigma or ignorance of transgender health issues from providers [12,13]. Further, transgender people are at significantly elevated risk of suicide ideation and attempt [14,15].

It is imperative to better understand the nature of these experiences in order for public health and social work practitioners to implement effective intervention strategies. With notable exceptions [2,9], few studies have employed Web-based approaches to studying transgender populations. To our knowledge, no other studies have used online social media websites for such a purpose.

### Transgender Communities and Online Social Networks

The Internet, and particularly social media websites, have shaped the way that people from marginalized, and otherwise stigmatized, groups communicate by allowing them to organize in large numbers around common interests and goals [2]. Evidence suggests that sexual minority (lesbian, gay, bisexual) individuals are more likely to use social media technologies than heterosexual individuals [16]. Online social networks allow users a certain degree of anonymity, possibly allowing researchers access to population groups in which privacy and secrecy are important. This may include some transgender people who feel obligated to publically hide their gender identities out of fear, shame, and desire to change [1].

Prior qualitative work has demonstrated that men who have sex with men (MSM) willingly enrolled in an online social media-based intervention and voluntarily had conversations with each other about common areas of interest and perceived health needs. This work demonstrated the unique potential to understand this population through a social media-based approach [17]. Statistics on social media usage are not available for transgender communities, but youth report having little access to information on transgender health concerns, and that this type of knowledge is commonly sought through the Internet [7].

This study attempts to determine the feasibility of accessing communities of transgender individuals and allies through the online microblogging platform, Twitter. We performed a content analysis of tweets (messages) posted to several transgender-related hashtags (analogous to discussion boards) in order to classify the types of information being communicated, the ways in which they are communicated, and to gain a community-level perspective on the perceived health and social needs faced by transgender communities. This approach represents a novel, effective way of accessing information about the needs of a population that has traditionally been difficult to reach.

## Methods

### Institutional Review Board

This is an observational study, which used publically available data. As such, the study does not meet the definition of human subjects research and was exempted from review by the Office of the Human Research Protection Program at the University of California, Los Angeles (UCLA).

### Twitter

Twitter is a popular social networking platform that allows users to post brief messages, limited to 140 characters, called “tweets”. Tweets can be shared with other users in a number of ways. These include posting the message to the original tweeter’s or another user’s or organization’s home page, where it can also be viewed easily by all other users who view those pages. Users have the option to “follow” other users, automatically updating them whenever a change is made to the followed page. In addition, users may retweet (repost) tweets of interest and affix tweets with a “hashtag”, denoted with a “#” applied before a keyword (for example, “#transgender”). Hashtagging a tweet places the message on a communal discussion board specific to that hashtag. This function is especially useful for allowing users to organize around a topic of interest, by placing all tweets affixed with a given hashtag in a central location that is easily searchable [18].

### Data Collection and Processing

Tweets were collected through the applied use of hashtags. All tweets posted to 13 different transgender and gender nonconforming-specific hashtags were collected over a one-day period on July 11, 2014 (Table 1). Due to a software malfunction, only a subset of tweets was collected from the hashtag, “ftm”. Therefore, this hashtag was excluded from analysis. Due to the large number of tweets made daily to these hashtags, one 24-hour period was considered sufficient for this feasibility study. A thorough Internet search was performed to identify hashtags that were currently in use, and designed for transgender community usage. Given the rapidly evolving nature by which social media platforms are used, as well as the identifying terminology used by transgender populations, only hashtags that appeared to be specific to transgender communities on the date of collection were selected for inclusion in the study.

Tweets were collected using the Twitter Archiving Google Spreadsheet (TAGS) version 5, a programmable Google Spreadsheet developed for time-based tweet collection [18,19]. The spreadsheet was programmed to collect all new tweets posted to an individual hashtag on an hourly basis. There were 5860 tweets that were collected in total. After compiling all tweets and removing duplicates (occurred when multiple hashtags were applied to a single tweet, resulting in the TAGS software collecting the same tweet from both hashtags), 5454 tweets remained. Despite the consideration with which hashtags were chosen for inclusion in the study, a considerable number of tweets irrelevant to transgender communities were posted to the hashtags, and 3161 such tweets were removed from analysis. Finally, non-English language tweets, and tweets containing sexually explicit material were also removed, resulting in a final dataset of 1135 analyzed tweets. Tweets containing sexual health-related text were relevant to this analysis, and so were left in the dataset.

**Table 1:** Hashtags from which tweets were collected.

Hashtags	Total	Proportion of total
#trans	507	0.45
#transgender	457	0.40
#girlslikeus	161	0.14
#mtf	47	0.04
#genderqueer	14	0.01
#nonbinary	12	0.01
#genderbender	11	0.01
#f2m	5	0.00
#m2f	2	0.00
#transguy	2	0.00
#transproblems	2	0.00
#dysphoria	1	0.00
#transgirl	1	0.00

### Data Analysis

A content analysis was performed. Tweets were coded and analyzed in a Web-based mixed-methods data analysis software, Dedoose [20]. Tweets were coded along multiple dimensions: (1) hashtag(s) from which a tweet originated, (2) general themes or topics addressed within the message, and (3) whether or not information was shared in a distinctive way (whether the tweet was an original post or a retweet, whether a link to a relevant news article was included in the message, and whether the message was specifically posted on an organization’s home page).

Codes were developed as general themes emerged from the data, and were assigned to all tweets to which they applied. The coding process, completed by a single researcher with expertise in transgender topics, was completed twice. No new codes were applied during this second review of the data; the purpose was to ensure that all tweets were coded in a consistent way. Care was taken to code tweets according to the text presented within them, and to avoid inferring meaning as much as possible. As such, it was possible for different tweets written about the same issue to be coded in slightly different ways. Table 2 shows examples of tweets, as they were coded.

**Table 2:** Topics discussed among transgender and gender nonconforming individuals and allies on transgender-specific hashtags on Twitter.

Category	Example tweet	Codes applied^a^
Social (positive)	Atherton High School #Kentucky Finalizes #Anti-Discrimination Policies For #Transgender Students [link to news article] via @wfplnews Nice!	6
	we are all #equal === sending my love to #gay #lesbian #bisexual #transgender peeps xoxoxo … #LGBTPrideMonth #equal #rights #marriage	3, 23
Social (negative)	Hobby Lobby takes stand against “Transgender Restrooms” #Transgender #HobbyLobby [link to news article]	11
	W/out accurate ID #transgender people face tremendous difficulty fully participating in society, finding jobs or accessing benefits & services	12, 15, 18
Personal (positive)	One year hrt #transgender #transpride #over40 #happy [link to image]	23, 24
	Last wknd in "boy mode" around my kids. Finally coming out to them as my authentic self later this month #transgender [link to image]	24
Personal (negative)	The worst part about having a female body is the fact that when I swim I have to wear a top #transgender #ftm #swimming #bathingsuit	27
	RT @cns_health: Press Conf #AIDS2014: #MSM & #transgender need to protect themselves from anal STIs & #HIV [link to article]	25

^a^ Codes were applied as numbered in Table 3

## Results

### Hashtags Applied to Tweets

Hashtags were applied 1222 times to 1135 independent tweets. Further, the following three hashtags represented 92.06% (1125/1222) of the total hashtags applied: “#transgender”, “#trans”, and “#girlslikeus” (Table 1). There were 94.45% (1072/1135) of tweets that addressed topical areas that fell into at least one of four major categories, each containing several subcategories, applied as codes: “social (positive)”, “social (negative)”, personal (positive)”, and “personal (negative)” (Table 3).

### Contents of Tweets

Social (positive) tweets were represented by messages that described socially progressive ideas, events, or actions taken by others beyond the original tweeter. Commonly occurring themes in this category included messages of ally affirmation or support for transgender individuals and causes, discussions around improving social conditions for transgender individuals, and messages related to research conducted that was specific to transgender needs. Social (positive) tweets accounted for 54.71% (621/1135) of all tweets in the dataset. Social (negative) tweets accounted for 26.34% (299/1135) of all tweets, and were characterized by messages related to socially conservative ideas, or actions that harm transgender individuals or communities. Examples of such themes include descriptions of discriminatory policies, violence experienced by transgender individuals, or accounts of ignorance about transgender-specific issues. Personal (positive) tweets, accounting for 11.10% (126/1135) of all tweets, included messages of self-affirmation, self-affirming comments regarding one’s physical appearance, or declarations of pride. Personal (negative) tweets accounted for 2.29% (26/1135) of all tweets, and included messages related to body image dysphoria, prior suicide attempts, and sexual risk behaviors.

There were five current events pertinent to transgender communities that were heavily discussed, accounting for 34.36% (390/1135) of all tweets analyzed, and were included in the major categories described above. Laverne Cox, a transgender actress and activist had recently been nominated for an Emmy award for her role in a television series, accounting for 19.82% (225/1135) of all tweets. Tweets related to Sparkle, a transgender pride festival held annually in July in Manchester, United Kingdom accounted for 4.58% (52/1135) of tweets analyzed. An Internet video of a Fox News clip, in which interviewees failed to become upset upon learning about a local gender inclusive restroom policy was also shared in 0.70% (8/1135) of tweets. Finally, information was shared about two organizations’ discriminatory policies, both having recently garnered media attention. George Fox University, a Christian university, had recently been granted a Title IX exemption allowing the university to deny gender-appropriate housing to transgender students, accounting for 5.81% (66/1135) of tweets. Hobby Lobby, a popular chain craft store, was highlighted in news stories for refusing a transgender employee access to a gender-appropriate restroom, accounting for 3.44% (39/1135) of tweets.

Despite the positive or negative themes present within the tweets, the ideological orientations/attitudes of the individuals posting them were overwhelmingly positive and affirming of transgender communities. Of all tweets analyzed, four were coded as having come directly from individuals espousing clearly “negative” views toward transgender people, for example,

This "#Baptist" #Church thinks it's OK to have a #Transgender pastor. (link to news article) - Satan has hijacked the Baptist name!

In addition, 60.79% (690/1135) of tweets contained a URL linking the reader to a news article or resource directly related to the central message of the tweet. Retweets consisted of 46.43% (527/1135) of tweets, whereby a user copies and reposts a tweet originating from another user. Finally, 33.13% (376/1135) of tweets linked the message text to an organization’s Twitter account.

**Table 3:** Four major categories of conversation, each consisting of several subcategories.

Categories^a^		Total	Proportion of total
**Social (positive)**			621/1135 (54.71%)
	1. Laverne Cox Emmy nomination	225	0.20
	2. Advertisement of an event, service, or job/solicitation of help at an event/on a project	120	0.11
	3. Ally affirmation/support	84	0.07
	4. Sparkle festival	52	0.05
	5. Transgender celebrity news (not including Laverne Cox Emmy nomination)	48	0.04
	6. Descriptions of improvements in social condition for transgender individuals	36	0.03
	7. Transgender-specific research findings	31	0.03
	8. Recognition of correct gender identity on legal documents	17	0.01
	9. Video of a Fox News interview, depicting interviewees who failed to become upset about enactment of a gender inclusive restroom policy	8	0.01
**Social (negative)**			299/1135 (26.34%)
	10. George Fox University (housing discrimination)	66	0.06
	11. Hobby Lobby discriminatory restroom policy	39	0.03
	12. Discrimination (general)	37	0.03
	13. Violence	31	0.03
	14. Lack of understanding, ignorance toward transgender issues or individuals	29	0.03
	15. Workplace discrimination	24	0.02
	16. Descriptions of other individuals holding negative views toward transgender people^b^	24	0.02
	17. Police mistreatment or brutality	17	0.01
	18. Refusal to recognize one’s gender identity, either interpersonally or legally	13	0.01
	19. Discriminatory treatment of prison inmates	8	0.01
	20. Tokenizing of transgender population, generalizations applied to whole population	6	0.01
	21. Insulting remark about transgender celebrity	5	0.00
**Personal (positive)**			126/1135 (11.10%)
	22. Interest in appearance	51	0.04
	23. Pride	38	0.03
	24. Self affirmation	37	0.03
**Personal (negative)**			26/1135 (2.29%)
	25. Sexual risk behavior	10	0.01
	26. Suicide	7	0.01
	27. Body image dysphoria	3	0.00
	28. Depression	3	0.00
	29. Eating disorders	3	0.00

^a^ Topics presented represent major categories and themes of conversation presented within tweets. Individual messages marked with a specific code, for example, violence, may not pertain to the same event.

^b^ Does not indicate a negatively held view by the original tweeter; instead, indicates the negative views of others, as acknowledged within the text of a tweet.

## Discussion

### Online Social Media

Online social media provides users with platforms to interact and share information in increasingly innovative ways, and provides researchers with new points of access to information. Twitter is one such platform, in which short messages, limited to 140 characters, may be composed and shared with a wide audience. Additionally, such platforms allow users to share as much or as little personal information with other users as is desired. Users are able to create a username of their choice and omit additional identifying information. As such, Twitter provides a platform for discussions about controversial or taboo topics with relative anonymity, that many may be uncomfortable discussing in a less anonymous setting.

Information is shared commonly, and with relative ease among Twitter users. This is evidenced both by the large number of tweets providing links to news articles and the high rates of retweeting and posting of tweets to organizational Twitter accounts. Organizational pages may be seen as central locations in which Twitter users can easily find and access information. The high degree of information sharing and the methods of sharing employed by users may be of importance to consider when designing effective community-based prevention strategies. Online prevention strategies, specifically those delivered through social networking websites, may benefit by encouraging peer-to-peer information sharing.

### Transgender Support on Twitter

Transgender individuals and allies use Twitter to discuss a large range of topics relevant to the social conditions they experience. Additionally, over one-third of tweets addressed relevant current events, demonstrating that Twitter provides a platform for sharing news about such topics with other members of the community in real-time, and potentially for mobilizing around an issue of interest. It is important, and rather interesting to note, the disproportionately large number of “positive” posts over “negative” ones. While it is possible that the ratio of positive to negative tweets is unique to the date on which data were collected (July 11, 2014), or to the specific hashtags surveyed, it may also be indicative of the purpose that Twitter serves to transgender individuals and allies, as a place of camaraderie and support. The finding that only 4 tweets were posted by users who openly espoused negative views of transgender communities further supports this notion. Indeed, while many tweets certainly did address the very real health and social issues faced by transgender communities, the Twitter hashtags surveyed appear to serve as a place of optimism for social progress.

### Limitations

This method of data collection may represent a novel, effective way of accessing information about a population that has traditionally been difficult to reach. However, our study presents several limitations to researchers. First, and perhaps most limiting, tweeters utilize the platform in an uncontrolled manner. They are free to post messages to the hashtags from which data are collected, to other hashtags, or to no hashtag at all. Indeed, a substantial number of tweets initially collected were dropped from analysis because they were irrelevant to transgender issues. The nature of this research is purely observational, and so significant data cleaning and processing was necessary to abstract relevant information. Further, without careful selection of hashtags from which to collect tweets, large portions of relevant data, and potentially from distinct demographic subgroups, may go uncollected. Additionally, tweets are written and posted on a voluntary basis. Our dataset includes tweets from users who were interested in talking about transgender-related issues on the date of collection, and is not specific to transgender-identified individuals. However, given the overwhelmingly “positive” natures of those who posted, it is clear that this sample includes largely transgender-identified individuals and allies.

Second, given the relative anonymity of such networks, it is impossible to obtain accurate demographic information on those being studied. As such, we do not know the ages, races, ethnicities, educational, or employment backgrounds, or even the identified genders of participants, unless explicitly stated by the user. Additionally, only tweets written in English, and on the single day of data collection are represented in this sample. These findings may not remain stable, as pertinent issues vary over time, and across different language and cultural groups. While Twitter may represent a novel approach to understanding transgender-related needs, it is not able to provide representative population estimates. Instead, it is able to provide meaningful and real-time information about a specific population of people, Twitter-using individuals who are interested in transgender-related topics.

Third, we attempted to access tweets from a wide range of transgender-related hashtags. As such, our results paint a global picture of transgender-related issues. Given the heterogeneity of individuals and communities that qualify as “transgender”, it is important to recognize that despite some common experiences, different subpopulations will likely communicate different needs and concerns. Future studies should attempt to disentangle the unique needs of specific transgender communities, such as male-to-female, female-to-male, and gender nonconforming populations.

Finally, social acceptance and usage of different social networking platforms may vary over time. The ways in which users utilize a platform (eg, for political discourse vs socializing) will directly affect the data that are abstracted [21]. Researchers will need to keep abreast of such changes in order to effectively collect data. However, the approach demonstrated here provides an innovative means of understanding the unique needs of an understudied population, both cheaply and in a timely manner [22].

## Abbreviations

MSM: men who have sex with men
TAGS: Twitter Archiving Google Spreadsheet
UCLA: University of California, Los Angeles
